# Proteomic survey of the *Streptomyces coelicolor* nucleoid

**DOI:** 10.1016/j.jprot.2013.02.033

**Published:** 2013-05-27

**Authors:** Elizabeth Bradshaw, Gerhard Saalbach, Michael McArthur

**Affiliations:** John Innes Centre, Norwich, UK

**Keywords:** GR, Global regulator, NAP, Nucleoid-associated proteins, DNA-binding, LC–MS/MS, Nucleoid, H-NS, IHF, *Streptomyces*

## Abstract

Nucleoid-associated proteins (NAPs) are small, highly abundant transcriptional regulators with low sequence specificity which are involved in multiple DNA-related processes including gene expression, DNA protection, recombination/repair and nucleoid structuring. Through these functions they are able to regulate important phenotypic properties including virulence, secondary metabolism and stress resistance. However the set of NAPs known within the Actinobacteria is small and incomplete. The missing proteins are likely to be key regulators of virulence in pathogens such as *Mycobacterium tuberculosis* and also of development and secondary metabolism in industrially-important species such as *Streptomyces*. Here, we use label-free LC–MS/MS to systematically search for novel NAPs in isolated nucleoids of the model actinomycete *Streptomyces coelicolor*. Based on the criteria of high abundance (emPAI score) and predicted DNA-binding ability (DNAbinder score) we identified a set of 24 proteins with a high predicted likelihood of being NAPs. The approach was deemed successful as the set included known major NAPs HupA, HupS, sIHF and Lsr2 as well as the global transcriptional regulators BldD and CRP and the pleiotropic response regulator AfsQ1. It also included a number of proteins whose functions are not yet known from recognisable classes of transcription factor (SCO2140, SCO4493, SCO1839, SCO1210, SCO5405, SCO4229, SCO3198) or from uncharacterised protein families (SCO5783, SCO5592, SCO3793, SCO6482) which comprise a valuable set of candidates for further study.

**Biological significance:**

In this paper we establish a robust protocol for preparing *S. coelicolor* nucleoids for mass spectrometric analysis and develop a workflow for identifying novel nucleoid-associated proteins (NAPs) by combining LC–MS/MS with a bioinformatical analysis. The nucleoid-associated proteins of many species are known to be key regulators of virulence, stress tolerance and global patterns of gene expression. Identifying the “missing” nucleoid proteins of *S. coelicolor* is likely to have important implications for manipulating the production of secondary metabolites such as antibiotics. Candidate NAPs were identified. Several of these are highly conserved in clinically important species such as *Mycobacterium* and in many commercially important species such as *Salinispora* and *Micromonospora* which represent a vital source of novel drugs such as antibiotics, antifungals and anticancer agents.

## Highlights

• *Streptomyces coelicolor* was grown in liquid culture to late vegetative phase.
• Whole nucleoids were isolated by sucrose gradient sedimentation.
• Proteins attached to the nucleoids were identified by label-free LC–MS/MS.
• A list of high-abundance DNA-binding proteins was generated, representing likely NAPs.

## Graphical abstract

**Figure:**
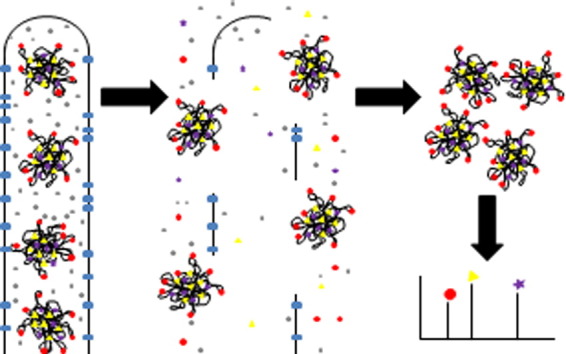

## 1 Introduction

The bacterial nucleoid is a non-membrane-bound region in the middle of the cell which contains the chromosome and its associated protein and RNA species. Nucleoid-associated proteins (NAPs) are generally defined as small, highly abundant DNA-binding proteins with low sequence specificity which have widespread effects on both gene expression and chromatin structure [1]. They will typically be present in tens of thousands of monomers per cell and affect the expression of 5–10% of the genome [2–4]. Proteins such as cAMP receptor protein (CRP) may be described as global regulators (GRs) since they have very large regulons [5] but have not been shown to substantially contribute towards nucleoid structure. NAPs are a highly heterogeneous group, representing a variety of protein folds and mechanisms of action. Some are involved in DNA repair (e.g. HU, [6]) or post-transcriptional processes (e.g. StpA, [7]) in addition to roles in nucleoid structure and the global transcriptional programme. Other NAPs such as Dps may have minimal roles in regulating gene expression but are involved in physical and chemical protection of the chromosome, with some homologs (e.g. from *Agrobacterium tumifaciens*) having lost the ability to bind DNA over the course of evolution [8]. In addition to a “core set” of around 10–20 NAPs and GRs the nucleoid also contains many dozens of low-abundance local transcription factors, as well as proteins which interact indirectly with the nucleoid by protein–protein interactions and large molecular machines or enzymes which act on DNA (polymerases, helicases, DNA methyltransferases).

NAPs are commonly involved in regulation of clusters of genes acquired by horizontal gene transfer, such as pathogenicity islands [9]. We hypothesize that NAPs will similarly be important in Actinomycetes for regulation of genes in secondary metabolic clusters, whose expression levels are also specific to certain growth conditions or developmental stages and may be found in genomic islands [10]. The aim of this study was to identify novel NAPs/GRs in *Streptomyces coelicolor* which could subsequently be tested for pleiotropic effects on secondary metabolism, in particular on expression from cryptic clusters for which no product has been observed.

The majority of research on NAPs has been carried out in members of the Enterobacteriaceae but these proteins are often absent, altered or duplicated in other lineages. A number have been identified in the *S. coelicolor* genome by sequence similarity with known NAPs from other species but more remain to be found. The number remaining to be found in *Streptomyces* is not known, but may be higher than in other species as it has a large genome with a particularly high proportion of sigma factors and transcriptional regulators [11]. Two copies of HU are present, the vegetative version of which resembles *Escherichia coli* HU and the spore-associated version of which uses a novel lysine-rich tail to compact and protect DNA specifically in aerial mycelium and spores [12,13]. Two copies of the H-NS-like protein Lsr2 and one copy of sIHF [14], both originally identified in *Mycobacterium*, are present but not yet well characterised. Multiple genes encoding transcription factors annotated as belonging to the Lrp/AsnC family can be found but it is not known whether any of these are truly global regulators while equivalents of Fis and Hfq have never been identified.

Discovery of novel NAPs is not trivial as conventional genetic screens are not possible due to the absence of a readily-screenable phenotype. Many of the canonical NAPs of *E. coli* were originally discovered during studies on bacteriophages, for example IHF (integration host factor), Fis (factor for inversion stimulation) and Hfq (host factor for phage Qβ). Therefore these constitute an important set of proteins which are difficult to identify on the basis of shared structure of common function. Instead, a direct biochemical approach must be taken. We reasoned that the most direct approach would be to survey the protein content of intact nucleoids which are obtained by gentle lysis of cells into a buffer containing spermidine and a physiological salt concentration, then recovered by sucrose gradient centrifugation. Nucleoids isolated in this manner from other species have previously been shown to contain large quantities of NAPs such as HU/H-NS/Fis and also co-sediment with post-transcriptional regulators of gene expression such as the global RNA chaperone Hfq and the translation apparatus [15,16]. A protocol for isolating intact nucleoids from *Streptomyces hygroscopicus* had previously been described by Sarfert et al. [17] but they were unable to identify the proteins present.

In this paper we use label-free LC–MS/MS to identify the abundant proteins found in the nucleoid of the model Actinomycete *S. coelicolor* and a bioinformatic analysis was performed to determine which of these were most likely to be NAPs, generating a list of candidates for further study. It is not possible to separate NAPs from global regulators from these data as effects on chromatin structure are not measured, therefore they will be considered together here.

## 2 Materials and methods

### 2.1 Experimental design

In order to study a given subcellular fraction, a comparison is commonly made between its protein composition and that of a whole-cell lysate or dissimilar fraction. However this approach is not necessarily appropriate when studying NAPs because of their great abundance in such a large, loosely-defined cellular structure and because the amounts of unbound NAPs normally present in the cytoplasmic “pool” are unknown. In a similar study by Ohniwa et al. [15], suspected contaminants were subtracted by comparing the composition of the nucleoids against cell envelope and “top matter” fractions (material remaining at the top of a preparative sucrose gradient following ultra-centrifugation), then classifying proteins which were at least three times more abundant in the nucleoid fractions than the others as “csNAPs” (contaminant-subtracted NAPs). While this removed a proportion of the probable contaminants, such as porins and metabolic enzymes, this method excluded the major NAPs H-NS and Hfq. In this study we aimed to identify promising candidate NAPs so we chose not to compare the nucleoid samples with other fractions (a “ruling out” approach) but rather to prioritise the most promising candidates from the lists of detected proteins as they stand (a “ruling in” approach).

### 2.2 Bacterial strains and growth conditions

Spore stocks of *S. coelicolor* A3(2) M145 were inoculated into the rich liquid medium TSB:YEME34%, made by mixing equal volumes of tryptone soya broth and YEME34% (yeast extract, malt extract, 34% sucrose) [18], in baffled flasks and incubated at 30 °C with constant shaking (225 rpm) until the cultures reached the late vegetative phase of growth. Growth was measured by reading the OD450 of well-mixed culture on a Thermo Spectronic BioMate3 spectrophotometer, using 50/50 TSB:YEME34% as a blank. Undecylprodigiosin concentration was measured by resuspending 500 μl culture in an equal volume of acidified methanol and centrifuging again briefly (to pellet the debris), then measuring the absorbance of the supernatant at 530 nm against a blank of acidified methanol. Total actinorhodin concentration was measured by mixing 660 μl culture with 330 μl 2 M KOH, vortexing for 10 s, then centrifuging briefly to pellet the debris. The absorbance of the resulting supernatant was measured at 640 nm against a blank of 660 μl TSB:YEME34% mixed with 330 μl 2 M KOH. The growth phases were defined as follows: mid-log phase at around 25 h post-inoculation when mycelium was pale and OD_430_ was around 6; transition phase at 44 h post-inoculation when mycelium was bright red and OD_430_ was around 12; stationary phase from 70 h post-inoculation when mycelium was dark blue = purple and OD_430_ was around 15. A representative growth curve showing OD_430_, undecylprodigiosin production and actinorhodin production is provided in the supplementary materials.

### 2.3 Nucleoid isolation and protein recovery

50 ml mycelium was harvested by centrifugation at 3000 ×*g* for 5 min at 4 °C in a Sorvall GS3 rotor then washed once in 50 ml Buffer 1 (10 mM Tris–HCl pH 8, 10 mM NaCl, 20% sucrose). This was centrifuged as before and resuspended in 5 ml Buffer 1 containing one tablet of complete mini EDTA-free protease inhibitor cocktail (Roche). 50 μl of Buffer 2 (10 mM Tris–HCl pH 8, 50 mM EDTA, 10 mg/ml lysozyme) was added and the mixture was incubated at 4 °C for 90 s. 5 ml Buffer 3 (10 mM EDTA, 1% Brij58, 0.4% sodium deoxycholate, 12 mM spermine) was added and the mixture was incubated for 20 min at 10 °C with occasional gentle inversion. The NaCl concentration was then adjusted to 200 mM and the mixture was incubated at room temperature for a further 10 min until slight visible clearing had occurred, indicating lysis. 4 ml of lysate was layered on top of a 24 ml 10–30% linear sucrose gradient and centrifuged at 10,000 ×*g* for 60 min in a Sorvall SS-34 rotor. 1 ml fractions were collected from the top of the gradient using a cut-off 1 ml pipette tip. The DNA concentration of each fraction was estimated with a NanoDrop ND-1000 spectrophotometer (NanoDrop Technologies, DE) and the most DNA-rich fraction was taken forwards for LC–MS/MS. 1D SDS-PAGE analysis showed that the protein content between different DNA-rich fractions was similar (not shown), therefore only one fraction was analysed in each experiment. Fractions were concentrated by methanol precipitation then run a short distance into a RunBlue 16% SDS-PAGE gel (Expedeon). In order to reduce the complexity of the samples and exclude HMW proteins, three gel slices were cut from each lane covering 5–11 kDa, 11–13 kDa and 13–31 kDa respectively.

### 2.4 ESI-Orbitrap MS/MS

After in-gel trypsin digestion, peptides were extracted with 5% formic acid/50% acetonitrile, dried down, and re-dissolved in 0.1% TFA. For LC-MS/MS analysis, a sample aliquot was applied via a nanoAcquity™ (Waters, Manchester, UK) UPLC™-system running at a flow rate of 250 nL min^− 1^ to an LTQ-Orbitrap™ mass spectrometer (Thermo Fisher, Waltham, MA). Peptides were trapped using a pre-column (Symmetry ® C18, 5 μm, 180 μm × 20 mm, Waters) which was then switched in-line to an analytical column (BEH C18,1.7 μm, 75 μm × 250 mm, Waters) for separation. Peptides were eluted with a gradient of 3–38% acetonitrile in water/0.1% formic acid at a rate of 0.67% min^− 1^. The column was connected to a 10 μm SilicaTip™ nanospray emitter (New Objective, Woburn, MA, USA) attached to a nanospray interface (Proxeon, Odense, Denmark) for infusion into the mass spectrometer. The mass spectrometer was operated in positive ion mode at a capillary temperature of 200 °C. The source voltage and focusing voltages were tuned for the transmission of MRFA peptide (m/z 524) (Sigma-Aldrich, St. Louis, MO). Data dependent analysis was carried out in oribtrap-IT parallel mode using CID fragmentation on the 6 most abundant ions in each cycle. The orbitrap was run with a resolution of 30,000 over the MS range from m/z 350 to m/z 1800 and an MS target of 10^6^ and 1 s maximum scan time. Collision energy was 35, and an isolation width of 2 was used. Only mono-isotopic 2 + and 3 + charged precursors were selected for MS2. The MS2 was triggered by a minimal signal of 1000 with an AGC target of 3x10^4^ ions and 150 ms scan time using the chromatography function for peak apex detection. Dynamic exclusion was set to 1 count and 30 s exclusion with an exclusion mass window of ± 20 ppm. MS scans were saved in profile mode while MSMS scans were saved in centroid mode.

### 2.5 Proteomics data analysis

Raw files obtained with the LTQ-Orbitrap™ mass spectrometer were processed with MaxQuant version 1.2.2.5 ([19]; http://maxquant.org) to generate re-calibrated pkl-files which were used for a database search using an in-house Mascot 2.3 Server (Matrixscience, London, UK). Searches were performed on the SPTrEMBL database (sptrembl20111116) with taxonomy set to *S. coelicolor* (8371 sequences) using trypsin/P cleavage with 2 missed cleavages, 6 p.p.m. precursor tolerance, 0.5 Da fragment tolerance, carbamidomethylation (C) as fixed, and oxidation (M) and acetylation (N-terminus) as variable modifications. The pkl-files from the three gel slices of each sample were combined in a merged Mascot search including a decoy database search. Mascot results were exported using a peptide expect value threshold of 0.05 and a significance threshold of 0.01 resulting in a false discovery rate (FDR) of 1.30% for replicate 1 and 1.17% replicate 2. The data (raw files and Mascot dat files) is available in the PRIDE database [20] (www.ebi.ac.uk/pride) under accession numbers 27029–27034 (username: review91665, password: RBKr_Mf). The data was converted using PRIDE Converter [21] (http://code.google.com/p/pride-converter).

## 3 Results and discussion

### 3.1 Experimental system

**Fig. 1:**
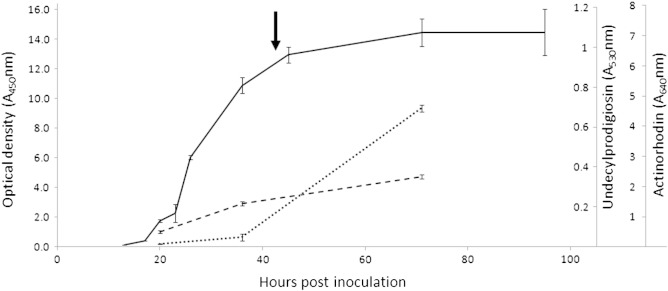
Growth curve of a typical *S. coelicolor* in rich liquid medium. Solid line = optical density (A_450_nm); dashed line = undecylprodigiosin production (A_530_nm); dotted line = total actinorhodin production (A_640_nm). Arrow indicates the time point used in this study (44 h after inoculation).

In order to minimise the noise generated by morphological differentiation and maximise the likelihood of identifying NAPs which will be relevant to the industrial fermentation of secondary metabolites such as antibiotics or antifungals, these experiments were carried out using liquid cultures because *S. coelicolor* does not sporulate in liquid culture. A growth curve showing optical density, undecylprodigiosin production and actinorhodin production are shown in Fig. 1. Two independent biological replicates were performed.

### 3.2 Nucleoid isolation

**Fig. 2:**
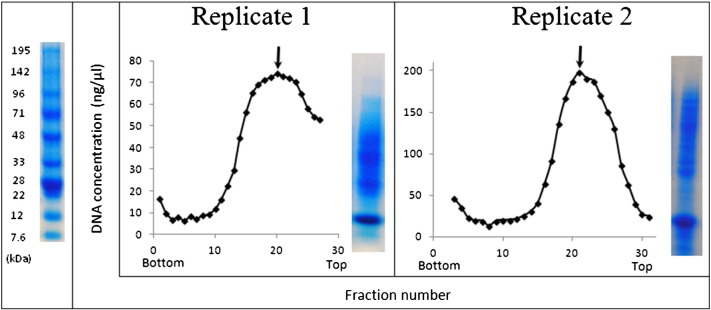
Sucrose gradient profiles and 1D SDS-PAGE gels of nucleoids harvested from two independent cultures: replicate 1 (left) and replicate 2 (right). Fraction numbers are displayed on the horizontal axis, from fraction 0 (bottom of the gradient) to fraction 30 (top of the gradient). Arrows indicate which fraction was harvested for both 1D SDS-PAGE and LC–MS/MS in each case.

Intact nucleoids were obtained by lysis of mycelium into spermidine buffer followed by sucrose gradient centrifugation, according to the method of Sarfert et al. [17]. Spermidine is a polyamine which stabilises the compact structures of isolated nucleoids by shielding negative charges on the DNA phosphate backbone [22,23]. The sucrose gradient profile (DNA content of sequential fractions collected from the gradient) was very similar to that obtained by Sarfert et al. [17], with a broad peak around the middle of the gradient and very little material retained at the top of the gradient (Fig. 2). Nucleoid-containing (DNA-rich) fractions were rich in high molecular weight (> 30 kb) DNA and RNA, with the position of the most DNA-rich fraction being responsive to brief digestion with RNaseI (moving upwards), demonstrating that RNA was contributing to the compaction of the nucleoids (not shown). The protein composition on 1D SDS-PAGE of the most DNA-rich fraction was similar to that observed by Sarfert et al. [17], with a predominant 12 kDa band (Fig. 2). The yield was significantly lower than that generally obtained from an equal volume of non-filamentous bacteria such as *E. coli*, suggesting that the process is less efficient in *Streptomyces*. This could be due to the morphology of *S. coelicolor*, which forms mycelial pellets in liquid into which penetration by lysozyme is likely to be limited over this timescale.

### 3.3 Protein identification and quantitation

Data files and lists of proteins are included in the supplementary materials. The total number of proteins identified in biological replicates 1 and 2 were 883 and 848 respectively. Of these proteins 14.7% and 14.4% respectively were annotated as known or putative transcriptional regulators, whereas experiments from a different group using whole-cell lysate analysed by 2DGE/MALDI-ToF found that known regulators made up only around 6% (47/770) of the species identified in whole-cell lysates [24], showing that enrichment for DNA-binding proteins had occurred. The true number of transcriptional regulators is likely to be slightly higher as some will not be annotated as such. The number of lipoproteins and outer membrane proteins was extremely low, showing that nucleoids had been cleanly separated from the cell envelope.

The method of quantification used in this study was the emPAI (Exponentially Modified Protein Abundance Index) score [25] which is based on peptide coverage. As with all quantification methods, artefacts are possible: saturation effects can be seen for highly abundant proteins, notably ribosomal proteins [25], but Ohniwa et al. [15] found that emPAI scores for NAPs such as HU, Dps and H-NS were correlated with the abundances determined by Azam et al. [26]. As these emPAI scores are not readily comparable between samples, both emPAI score and rank within the sample are given here when comparing quantities.

### 3.4 Proteins detected in the *Streptomyces* nucleoid

**Fig. 3:**
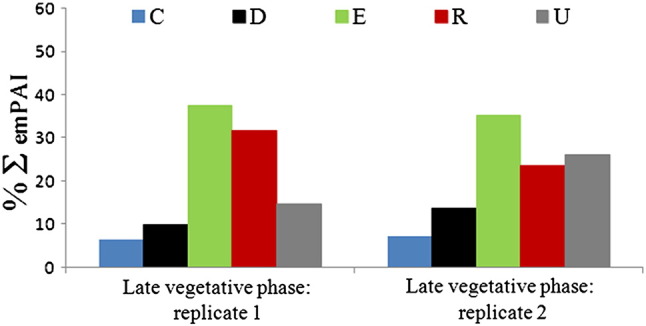
Classes of protein represented in the nucleoid fractions at each time point, by summed emPAI. Blue (C) = chaperones/cold-shock proteins; black (D) = DNA-/RNA-binding proteins; green (E) = enzymes/cytosolic function proteins/membrane proteins; red (R) = 30S/50S ribosomal proteins; grey (U) = proteins of unknown function.

Within this section rank refers to the position of a protein within the total list of proteins identified (supplementary data sheets “Total list replicate 1” and “Total list replicate 2”). All proteins were assigned to the following five functional categories based on their annotated function: 30S/50S ribosomal proteins and ribosome-associated proteins (R); chaperones, cold-shock proteins and redox-protective proteins (C); DNA-binding proteins (D); proteins of unknown function (U); metabolic enzymes, cytosolic proteins and membrane proteins (E). The abundances of proteins varied widely, with emPAI scores ranging from 31.16 down to 0.02, so that the protein composition within each replicate could not be readily calculated from the number of species in each category. As an approximate estimate of composition within each category, the summed emPAI scores of its members were calculated as a percentage of the sum of all emPAI scores at that time point (Fig. 3). Overall, the mixture of proteins was comparable in composition to that observed by Ohniwa et al. [15] from nucleoids of *E. coli*, *Bacillus subtilis* and *Pseudomonas aeruginosa*. In both replicates, a large proportion of the summed emPAI (32% and 24% respectively) was represented by ribosomal proteins, due to precipitation of RNA with the DNA of the nucleoids. The proportion of summed emPAI represented by enzymes and miscellaneous cytosolic proteins was 32% and 35% respectively, indicating a relatively high level of unavoidable contamination. The proportion represented by annotated DNA-binding proteins was 10% and 14% respectively, which was lower than the figure calculated simply from number of species because many of these proteins are of low abundance.

As previously observed by Ohniwa et al. [15], many proteins detected in nucleoid fractions were involved in redox chemistry, including multiple superoxide dismutases, thiol-specific antioxidant proteins and the alkyl hydroperoxide reductase AhpC [27]. The “armour hypothesis” suggests that these proteins are specifically associated with the nucleoid to protect the DNA from oxidative damage. Indeed, MnSOD of *E. coli* has been shown experimentally to associate non-specifically with DNA as a “tethered antioxidant” [28] and several well-characterised NAPs such as Dps and the HupS homolog Mdp1 have an additional redox function protecting DNA from Fenton chemistry. Tethered antioxidants are also known to be the present in mitochondrial and chloroplast nucleoids [29,30].

### 3.5 emPAI threshold for classifying annotated transcriptional regulators as NAPs/GRs

**Fig. 4:**
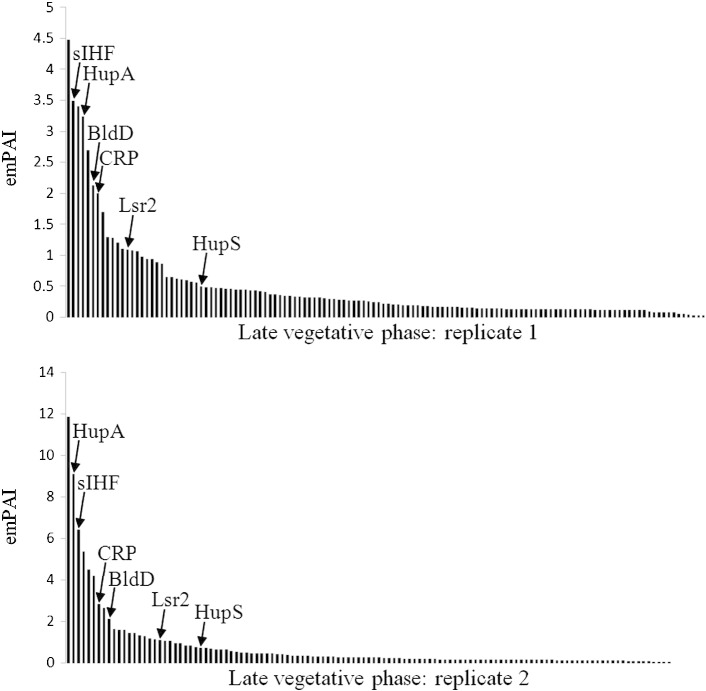
Annotated DNA-binding proteins (category D) ranked by abundance (emPAI). Known NAPs and GRs are labelled with arrows.

Of the proteins annotated as transcriptional regulators (category D) only a subset of these were anticipated to be NAPs or GRs but the expected number was not known. *E. coli* is generally reported to have around 10–20 NAPs but the list quoted varies between research groups, with no guarantee that all important *E. coli* NAPs have been discovered. The *Streptomyces* genome is particularly large and has a high proportion of transcriptional regulators in comparison to other species so it is possible that it also has more NAPs: gene duplication within this lineage has generated two paralogs of HU [12], two of Lsr2 and three of Dps [31]. In order to visualise the data more clearly, the emPAI scores for all proteins in category D were ranked and displayed as a column chart, with the positions of known NAPs marked by arrows (Fig. 4). It was apparent that there was no sound statistical basis for separating global from local regulators: rather than a pair of normally-distributed groups (one representing NAPs/GRs and one representing local regulators), the emPAI distribution was instead found to follow a power law, i.e. there are a small number of dominant species and a large number of low-abundance species which form a “long tail”, with no meaningful “typical” abundance for either group (Fig. 4). As expected, sIHF and HupA were extremely abundant in both replicates, being the second and third most abundant annotated DNA-binding proteins in both cases. The global regulators BldD and CRP were also very abundant, being in the top 10 most abundant proteins in both cases. Two other known NAPs, Lsr2 and HupS, were present in moderate amounts. Other important developmental regulators from the Bld, Whi and Wbl protein groups were detected at extremely low levels if at all, most likely because they are not strongly expressed in these non-sporulating cultures.

As there was not a clear distinction between NAPs and non-NAPs on the basis of abundance, a threshold of the top 30 category D proteins by abundance was taken to be potential NAPs as this group included HupA, HupS, sIHF, CRP, BldD and the Lsr2 protein SCO3375 in both replicates but excluded the majority of the long tail of low-abundance transcriptional regulators. Any proteins present in only one replicate were discarded from the analysis as possible contaminants.

### 3.6 Prediction of nucleic acid binding ability

A NAP may contain a recognisable DNA-binding domain such as a helix–turn–helix (e.g. Fis, Lrp) or alternatively may have a unique fold not shared by any local transcriptional regulators (e.g. HU, H-NS). Therefore some of the most interesting new NAPs are likely to have been categorised here as uncharacterised (U). All category U proteins with an emPAI score greater than that of the 30th most abundant protein in category D (DNA-binding) were screened for potential DNA-binding ability using *in silico* prediction tools. The helix–turn–helix prediction tool provided by NPS@ ([32]; available at http://npsa-pbil.ibcp.fr/cgi-bin/npsa_automat.pl?page=/NPSA/npsa_hth.html) did not identify any additional motifs which were not already present in the genome annotation. The online tool DNAbinder ([33]; available at http://www.imtech.res.in/raghava/dnabinder/index.html), was used to estimate the likelihood of binding from amino acid composition. For example proteins with a higher proportion of lysine, arginine and glutamic acid are predicted to be more likely to bind nucleic acids. The SVM model trained on the alternate dataset was selected as this was developed using full-length sequences. DNAbinder scores are included in the supplementary materials. It was not possible to classify any of the candidates further into DNA-binding and RNA-binding proteins because the prediction tools available are not sensitive enough to discriminate between the two. It is also plausible that some NAPs may bind both substrates, as does StpA [7]. Of the proteins from category (U) with a suitable emPAI score, 6 were found to have predicted DNA-binding ability and were added to the list of candidates.

### 3.7 Candidate NAPs/GRs

The preceding sections of this paper generated a list of 24 candidate NAPs/GRs which had high abundance and contained either a recognisable DNA-binding motif (18 proteins) or had an amino acid composition typical of nucleic acid binding proteins (6 proteins). The annotated class or function, TrEMBL accession number, SCO number, molecular weight, pI and rank at each time point for each protein are presented in Table 1. In this section rank refers to the position of a protein in this amalgamated list of category D and category U proteins.

The list of 24 candidates included the known NAPs HupA, HupS, Lsr2 and sIHF plus the known GRs BldD and CRP (Table 1). While independent confirmation of the candidates was beyond the scope of this study, the presence of 6 proteins which have been experimentally verified [12–14,34] strongly suggests that the approach was successful. The abundance of CRP supports the conclusion of Piette et al. [34] that it acts as a truly global regulator in *Streptomyces*. Rex (SCO3320), the redox-sensing repressor thought to work in concert with CRP [35], was not abundant at any time point.

The Lsr2 proteins were present in lower amounts than could have been expected for an H-NS equivalent. While SCO3375 (13th and 30th respectively) was abundant in both replicates, its abundance was significantly lower than those of sIHF or HupA. The other paralogue of Lsr2, SCO4076, was present at much lower levels (30th and undetected respectively). This could be due to inaccuracy in quantitation, or perhaps the proteins are not produced under the growth conditions tested, or there may be some functional redundancy with another H-NS-like protein present. *Mycobacterium* has one copy of Lsr2 alongside a second non-canonical H-NS protein which shares low sequence identity with *E. coli* H-NS [38] so it is possible that another as-yet-undiscovered H-NS-like protein exists in *Streptomyces*.

None of the three Dps proteins were detected at any time point during this study, suggesting that they do not have as strong a role in liquid cultures in this species as in *E. coli*. There is evidence that they have specialised to acquire roles in stress resistance and in cell division during sporulation [31,39].

The high abundances of the NAP HupS and the GR BldD were somewhat unexpected as these proteins are thought to play important roles in morphological differentiation [40–42], which does not happen in the liquid cultures used in this experiment. It is conceivable that they have secondary roles in non-sporulating cultures, for example during later growth phases or in the centres of mycelial pellets where stressful conditions occur. It would be interesting to test whether their abundance increases further in the nucleoids of cultures grown fully into stationary phase.

Eight of the candidates were annotated as transcriptional regulators based on the presence of DNA-binding motifs and similarity to known classes of locally-acting transcription factors. SCO3198, SCO4228 and SCO1210 are transcription factors of unknown function. The helix-turn-helix regulator SCO1839 (17th and 9th respectively) is very small (7.6 kDa) so presumably consists of a DNA-binding domain alone, with no ligand-binding domain. AfsQ1 is the response regulator of a two-component signalling system which is important in regulating both secondary metabolism and morphological development. SCO5405 is a response regulator which forms a two-gene operon with its cognate sensor kinase, SCO5404. Two proteins annotated as belonging to the AsnC family of transcription factors were also present, from which family the *E. coli* NAP Lrp also belongs. Inspection of the amino acid sequences of these (not shown) revealed that SCO4493 is a full-length proteins while SCO2140 is truncated, with the DNA-binding domain apparently being absent. It is conceivable that it nevertheless has a function in the nucleoid via protein–protein interactions, for example forming heterodimers with DNA-binding counterparts.

Two possible RNA-binding proteins were also on the list of 24 candidates. The highly-conserved but poorly-characterised RNA-binding host factor YajQ [36], which was previously reported as being required to support bacteriophage φ6 replication in *Pseudomonas syringae* [37], was abundant in both replicates (3rd and 4th respectively), however the function of this protein in chromosomal gene expression and nucleoid architecture is not clear. A second protein annotated as an RNA-binding protein of unknown function, SCO5592, was very abundant in both replicates (6th and 5th respectively). It is not known whether these were present in the samples due to association of RNA with isolated nucleoids or whether these proteins also have a DNA-binding role in this species.

Two proteins, SCO5783 and SCO6482, could not be confidently assigned to well-characterised classes of proteins despite extensive BLAST searches. These could represent either contaminants or NAPs belonging to an as-yet-unknown protein family.

Not all of the candidates identified will be genuine NAPs: any abundant contaminants with a reasonably high proportion of lysine and arginine will be score highly in this system. Several candidates were annotated with a function not previously associated with DNA-binding ability, including homologs of tellurium-resistance proteins such as SCO3767, which is a homolog of *Klebsiella* TerB whose solution structure demonstrated one very basic face and one very acidic face [43]. It is not immediately obvious how this would bind DNA or have NAP activity so it may simply be an abundant molecular chaperone. SCO5725 and SCO2129 are also unlikely to be genuine NAPs as they belong to the WGX-100 family of secretion-related proteins and the ARO-CYC family of enzymes respectively. YjqA (SCO3793) is a conserved homologue of the YjqA protein from *Bacillus subtilis*, whose function is unknown.

The list of candidate NAPs/GRs presented here is not intended to be an exhaustive catalogue of the contents of the *Streptomyces* nucleoid under all conditions. In particular these results may not accurately reflect the nucleoid composition of fully-differentiating cultures grown on solid media. There are also likely to be other NAPs induced by specific growth conditions not tested here, as is the case with YgiP from *E. coli* which is only seen under anaerobic conditions [44].

BLAST searches show that a number of the proteins (e.g. SCO5592,) identified as putative NAPs/GRs in this study are highly conserved in other Actinomycete species (not shown). If these are found to have significant functions in regulating secondary metabolism in *S. coelicolor* then they could represent a useful means of manipulating bioactive compound production to facilitate drug discovery in industrially-important species such as *Salinispora* and *Micromonospora*. Several of the putative NAPs/GRs are also present in pathogenic Actinomycetes such as *Mycobacterium*, *Nocardia* and *Actinomyces*, where they may play a role in virulence or stress-tolerance.

## 4 Conclusions

In this paper we used whole nucleoid isolation in combination with LC–MS/MS as a discovery tool for indentifying candidate novel NAPs/GRs, of which several are conserved within the Actinobacteria and may prove to be important regulators of pathogenesis, morphological differentiation or production of secondary metabolites such as antibiotics. To validate these candidates is well beyond the scope of the present study as it is not trivial to demonstrate that a protein is a NAP/GR: in addition to showing evidence of association with the nucleoid *in vivo* (for example by translational fusion to a fluorophore) there would also need to be evidence of a large regulon (~ 5–10% of the genome) and for classification as a NAP there would additionally need to be evidence that nucleoid or chromatin structure is affected at some level. However, there are a number of candidates worthy of further investigation, some of which we are pursuing, and we believe we have established a robust protocol which will be of use in future studies of the proteome of the nucleoid under different conditions.

## Figures and Tables

**Table 1:** The 24 most promising candidate NAPs/GRs found in *S. coelicolor*, arranged by their rank by abundance in replicate 1. Category D = proteins annotated as transcriptional regulators; category U = proteins annotated as uncharacterised. DNA binder scores are provided for category U (uncharacterised) proteins only; scores above zero indicate a high predicted likelihood of binding DNA or RNA. Asterisks indicate known NAPs/GRs which have been experimentally tested in other studies.

Name/function	NAPs/GRs	Category	SCO no.	TrEMBL accession	DNAbinder score	MW (kDa)	pI	Replicate 1 emPAI	Replicate 1 rank	Replicate 2 emPAI	Replicate 2 rank
AsnC-family regulator		D	2140	Q9X7Z9	n/a	10.1	4.8	4.47	1	4.47	9
sIHF	*	D	1480	Q9KXR9	n/a	11.5	10.4	3.49	2	6.4	7
YajQ		D	4614	Q9F2U7	n/a	18	5.5	3.4	3	9.02	4
HupA	*	D	2950	P0A3H5	n/a	9.9	9.5	3.24	4	9.1	3
Uncharacterised		U	5783	O69959	0.59	17.1	7.1	2.95	5	1.36	25
RNA-binding		D	5592	P0A4Q4	n/a	8.7	9.9	2.69	6	8.82	5
WXG-100		U	5725	O86644	1.66	11.5	5.2	2.49	7	2.49	14
TerB		U	3767	Q9F2L4	0.40	16.6	5.3	2.43	8	27.35	1
BldD	*	D	1489	O52732	n/a	18.2	6.6	2.12	9	2.12	15
CRP	*	D	3571	Q9XA42	n/a	24.6	6.2	1.99	10	2.81	11
YjqA		U	3793	Q9F325	1.00	13.6	7.1	1.91	11	7.48	8
RNA polymerase		D	4729	P60312	n/a	36.7	4.8	1.69	12	0.93	19
ARO/CYC		U	2129	Q9X7Y8	1.44	15.9	6	1.53	13	1.53	21
AsnC-family regulator		D	4493	Q9KYP0	n/a	17.9	5.6	1.28	14	0.93	20
SSBP		D	3907	Q9X8U3	n/a	19.9	5.4	1.1	15	0.81	23
Lsr2	*	D	3375	Q9X8N1	n/a	11.7	6.8	1.09	16	1.09	30
HtH regulator		D	1839	Q9RJ29	n/a	7.6	10.5	1.07	17	11.84	2
Uncharacterised		U	6482	Q9ZBJ2	0.03	16.1	5	0.73	18	1.5	22
Putative regulator		D	1210	Q9FCA3	n/a	23.9	6.7	0.65	19	0.65	24
Putative regulator		D	5405	Q9L2B5	n/a	18.3	6.7	0.62	20	1.62	18
AfsQ1		D	4907	Q04942	n/a	25.1	5.8	0.61	21	0.81	22
Putative regulator		D	4228	Q8CJU3	n/a	25.5	5.1	0.6	22	1.03	18
DeoR-family regulator		D	3198	Q9KYU7	n/a	27	5.9	0.56	23	1.44	15
HupS	*	D	5556	P0A3H7	n/a	22.3	11.2	0.49	24	0.71	24

## Appendix A Supplementary data

Supplementary material. Supplementary figure S1: (worksheet "fig. 1 growth curve") Data from which the growth curve shown in Figure 1 is derived. Supplementary figure S2: (worksheet "Total list replicate 1") Total list of proteins detected in nucleoid fractions in replicate 1 of this experiment. Supplementary figure S3: ("Total list replicate 2") Total list of proteins detected in nucleoid fractions in replicate 2 of this experiment. Supplementary figure S4: (worksheet "Figure 2") Data from which the growth curve shown in Figure 2 is derived. Supplementary figure S5: (worksheet "Figure 3") Data from which the growth curve shown in Figure 3 is derived. Supplementary figure S6: (worksheet "DNAbinder (U)") DNAbinder scores for all proteins detected at high abundance in nucloid fractions. Scores above the threshold for high likelihood of DNA-binding ability are shaded in green while those below the threshold are shaded in grey. Supplementary figure S7: (worksheet "D+U replicate 1") List of all proteins detected in nucleoid fractions at high abundance in replicate 1 of the experiment. Shading is as follows: black, category D; green, category U with DNAbinder score < 0; grey, category U with DNAbinder score > 0; red, proteins present in one replicate only. Supplementary figure S8: (worksheet "D+U replicate 2") List of all proteins detected in nucleoid fractions at high abundance in replicate 2 of the experiment. Shading is as follows: black, category D; green, category U with DNAbinder score < 0; grey, category U with DNAbinder score > 0; red, proteins present in one replicate only. Supplementary figure S9: (worksheet "Proteins for table 1") List of proteins from Table 1, with full functional annotation and DNAbinder scores shown. Supplementary figure S10: (worksheet "Table 1") Data from which the growth curve shown in Table 1 is derived.
